# CXCR4 Mediated Chemotaxis Is Regulated by 5T4 Oncofetal Glycoprotein in Mouse Embryonic Cells

**DOI:** 10.1371/journal.pone.0009982

**Published:** 2010-04-01

**Authors:** Thomas D. Southgate, Owen J. McGinn, Fernanda V. Castro, Andrzej J. Rutkowski, Mariam Al-Muftah, Georgi Marinov, Graeme J. Smethurst, David Shaw, Christopher M. Ward, Crispin J. Miller, Peter L. Stern

**Affiliations:** 1 Immunology Group, Paterson Institute for Cancer Research, University of Manchester, Manchester, United Kingdom; 2 Applied Computational Biology and Bioinformatics Group, Paterson Institute for Cancer Research, University of Manchester, Manchester, United Kingdom; Health Canada, Canada

## Abstract

5T4 oncofetal molecules are highly expressed during development and upregulated in cancer while showing only low levels in some adult tissues. Upregulation of 5T4 expression is a marker of loss of pluripotency in the early differentiation of embryonic stem (ES) cells and forms an integrated component of an epithelial-mesenchymal transition, a process important during embryonic development and metastatic spread of epithelial tumors. Investigation of the transcriptional changes in early ES differentiation showed upregulation of CXCL12 and down-regulation of a cell surface protease, CD26, which cleaves this chemokine. CXCL12 binds to the widely expressed CXCR4 and regulates key aspects of development, stem cell motility and tumour metastasis to tissues with high levels of CXCL12. We show that the 5T4 glycoprotein is required for optimal functional cell surface expression of the chemokine receptor CXCR4 and CXCL12 mediated chemotaxis in differentiating murine embryonic stem cells and embryo fibroblasts (MEF). Cell surface expression of 5T4 and CXCR4 molecules is co-localized in differentiating ES cells and MEF. By contrast, differentiating ES and MEF derived from 5T4 knockout (KO) mice show only intracellular CXCR4 expression but infection with adenovirus encoding mouse 5T4 restores CXCL12 chemotaxis and surface co-localization with 5T4 molecules. A series of chimeric constructs with interchanged domains of 5T4 and the glycoprotein CD44 were used to map the 5T4 sequences relevant for CXCR4 membrane expression and function in 5T4KO MEF. These data identified the 5T4 transmembrane domain as sufficient and necessary to enable CXCR4 cell surface expression and chemotaxis. Furthermore, some monoclonal antibodies against m5T4 can inhibit CXCL12 chemotaxis of differentiating ES cells and MEF which is not mediated by simple antigenic modulation. Collectively, these data support a molecular interaction of 5T4 and CXCR4 occurring at the cell surface which directly facilitates the biological response to CXCL12. The regulation of CXCR4 surface expression by 5T4 molecules is a novel means to control responses to the chemokine CXCL12 for example during embryogenesis but can also be selected to advantage the spread of a 5T4 positive tumor from its primary site.

## Introduction

5T4 oncofetal glycoprotein was discovered while searching for molecules with invasive properties likely to be shared by trophoblast and cancer cells [1]. It is expressed by many different carcinomas while showing only low levels in some normal tissues [2]. 5T4 expression has been shown to influence adhesion, cytoskeletal organization and motility [3], [4], [5], properties which might account for its association with poorer clinical outcome in some cancers [6], [7], [8], [9]. Its ≈72 kD transmembrane molecules have a short cytoplasmic region, as well as an N-glycosylated extracellular domain with two leucine rich repeat (LRR) regions separated by a hydrophilic sequence and associated N and C terminal flanking regions [10], [11]. LRR are found in proteins with diverse functions and are frequently associated with protein-protein interaction [12]. We have recently shown that upregulation of 5T4 expression is a marker of loss of pluripotency in the early differentiation of human and murine embryonic stem cells [13], [14] and forms an integrated component of an epithelial-mesenchymal transition (EMT) [15], [16]. EMT occurs during embryonic development and is also believed to be important for the metastatic spread of epithelial tumors [17]. To further study this process we conducted a comparative microarray analysis of undifferentiated (5T4 –ve) and early differentiating (5T4 +ve) murine ES cells [18]. 5T4 is up-regulated at an earlier stage of ES differentiation than the widely used down-regulation of the SSEA-1 marker [13] while cell sorting for surface 5T4 expression provided an additional level of stringency in the definition of ES cell populations compared to stratifications used in some other microarray studies [19], [20]. Any transcriptional changes may be important in governing the balance of self-renewal/pluripotency and differentiation in ES cells, or in the regulation of 5T4 cell surface expression. Such properties may also be functionally important in tumor progression. One significant transcriptional change identified was the down-regulation of transcripts for the dipeptidyl peptidase IV, CD26, which code for a cell surface protease that cleaves the chemokine CXCL12 [21]. Interestingly, differentiating ES cells also showed an upregulation of CXCL12 transcription. CXCL12 has been shown to regulate many biological processes but also plays an important role in tumorigenesis [22], [23]. CXCL12 binds to the widely expressed cell surface seven transmembrane domain G-protein coupled receptor CXCR4 [24], [25] and to the recently identified receptor CXCR7/RDC1 [26]. Upon ligand binding, CXCR4 undergoes a conformational change that facilitates activation of heterotrimeric G proteins and signaling effectors at the plasma membrane [27]. This initiates a signaling cascade resulting in downstream phosphorylation of proteins such as ERK1/2 and AKT [28], [29]. These activities are dependent on CXCR4 expression at the plasma membrane and cellular events that reduce the latter can abrogate the biological effects. Following activation, CXCR4 undergoes β-arrestin-mediated endocytotosis and although recycling of CXCR4 can occur this receptor can also be ubiquinated and directed to lysosomes where it is degraded [30], [31]. Both CXCL12 and CXCR4 expression have been associated with tumorigenesis in many cancers including breast, ovarian, renal, prostate, and neuroblastoma [22], [23], [24]. These CXCR4 expressing tumors preferentially spread to tissues that highly express CXCL12, including lung, liver, lymph nodes and bone marrow [22], [23], [24]. Therefore, the inverse correlation between 5T4 and CXCL12 with CD26 transcript levels during mouse ES cell differentiation, and the known roles of these molecules in cell migration/motility, may suggest that particular regulatory processes are common to both ES cell differentiation and tumor metastasis. This article reports the unexpected discovery that 5T4 molecules are required for functional expression of CXCR4 at the cell surface of differentiating ES cells, and mouse embryonic fibroblasts (MEF).

## Results

### Microarray analysis of differentiating mES cells stratified by cell surface 5T4 phenotype

Analysis of the microarray data from undifferentiated and differentiating E14 ES cells identified significant up or down regulation of transcripts in 148 and 277 genes respectively (GEO accession number GSE20372). There was a pattern of transcriptional changes relating to a loss of pluripotency in ES cells with significant (adjusted P value <0.1) downregulation of *Klf4* (18.4x), *Oct4* (1.6×) and *Sox2* (1.9×). Many genes known to bind these transcription factors and form part of the extended transcriptional network influencing pluripotency of ES cells were also found to be downregulated [32], [33]. These are approximately 25% of the significantly downregulated genes and include 77 which can bind one or more of these transcription factors. The complex changes in transcription seen in the differentiating ES cells analyzed here reflect the several different pathways that can control ES cell pluripotency and self-renewal. In respect of this report, the microarray data indicated a significant downregulation of *Dpp4* also called *Cd26* (6.2×), upregulation of *Cxcl12* (3.4×) but no change in the transcripts of *Cxcr4*. CD26 is a surface peptidase which cleaves CXCL12 chemokine and CXCR4 is the receptor of the latter. We decided to further investigate whether this was of functional significance.

### Differentiating mES cells show 5T4-dependent CXCL12 chemotaxis

**Figure 1:**
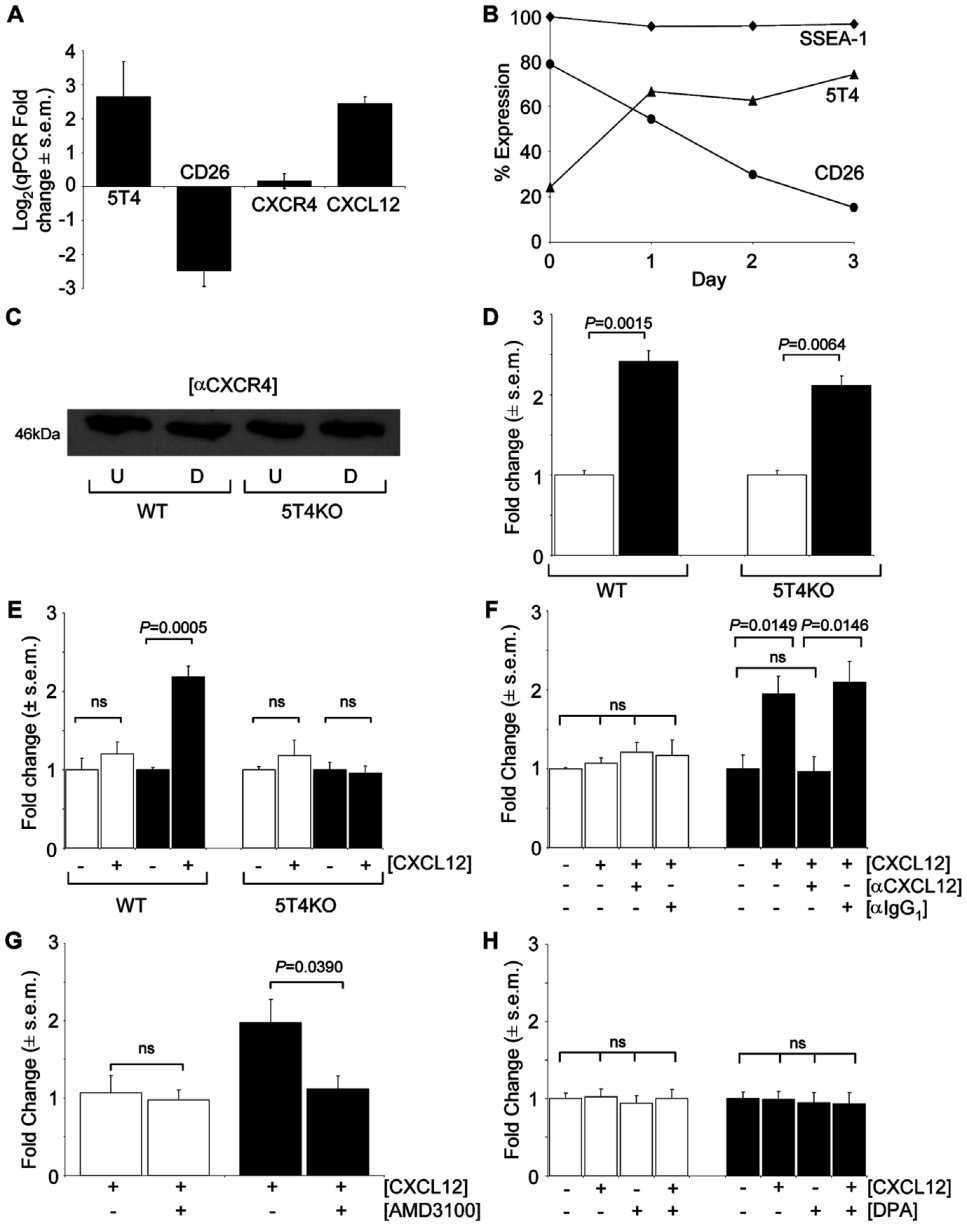
Differentiating mES cells show 5T4 dependent CXCL12 chemotaxis. (A), Triplicate quantitative RT-PCR of WT-ES cells after 3 days differentiation with significant changes in 5T4, CD26 and CXCL12 mRNA but not CXCR4 respectively *P* = 0.014, 0.057, 0.81, 0.012 by Student's t-test. (B), Flow cytometry analysis of WT-ES cell differentiation 5T4 (triangles), CD26 (circles) and SSEA-1 (diamonds) (n>3 a single representative time course shown). (C), Western blot analysis of PAGE separated reduced WT or 5T4KO-ES cells either undifferentiated (U) or differentiating (D) probed with CXCR4 antibody. (D), Murine CXCL12 specific ELISA of conditioned medium from undifferentiated (white columns) and differentiating (black columns) WT and 5T4KO-ES cells. (E), Undifferentiated WT and 5T4KO-ES cells (white columns) exhibit no CXCL12 chemotaxis. Differentiating (black columns) WT, but not 5T4KO-ES cells, acquire significant chemotaxis. (F), CXCL12 chemotaxis in differentiating WT-ES cells (black columns) is blocked by an antibody against CXCL12; undifferentiated ES cells (white columns) show no chemotaxis. (G), Chemotaxis of differentiating WT-ES cells, (black columns) is blocked by a 2hr pre-incubation with 10 µM AMD3100; with no effect on undifferentiated WT-ES cells (white columns). (H), Undifferentiated (white columns) or differentiating (black columns) 5T4KO-ES cells show no change in chemotactic response in the presence of the CD26 inhibitor diprotin A (DPA, 10 µM).

The latter microarray data were confirmed by qPCR (Figure 1A) and FACS analysis showed that as the ES cells differentiate cell surface expression of CD26 decreases while 5T4 increases; by contrast the pluripotent ES marker SSEA-1 did not significantly change over this time (Figure 1B). Consistent with the transcript analysis, western blotting shows there is no change in the total CXCR4 expression upon differentiation of either wild-type (WT) or 5T4 knock out (5T4KO)-ES cells (Figure 1C). A greater than 2 fold increase in CXCL12 was detected in the culture medium by ELISA after 3 days of differentiation of WT (71±4 vs 171±9 pg/ml) or 5T4KO (40±2 vs 84±5 pg/ml) ES cells, (Figure 1D). These data demonstrate that in early differentiation of ES ells there is increased CXCL12 ligand production and decreased expression of CD26 dipeptidase that can destroy its activity but there is no change in either the CXCR4 transcript or protein levels, consistent with the microarray results.

**Figure 2:**
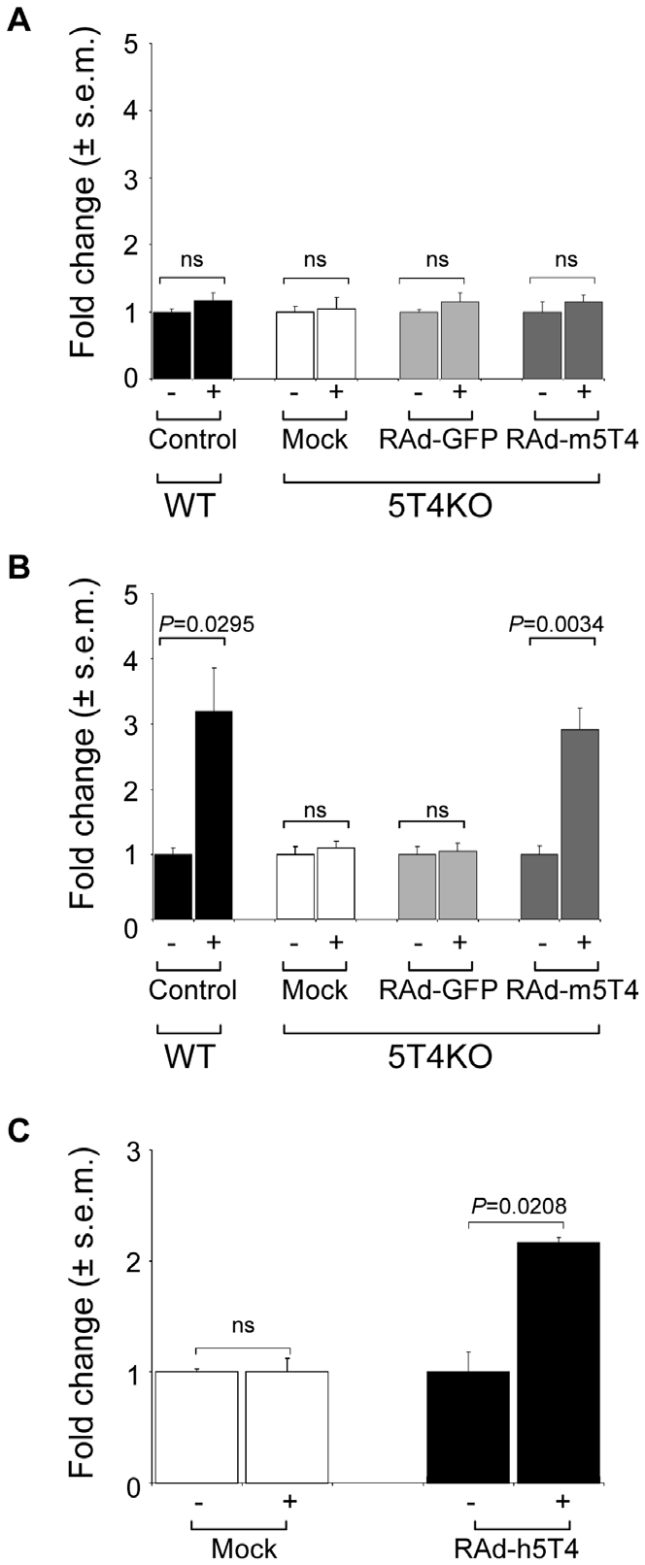
5T4 restores CXCL12 dependent chemotaxis in differentiating 5T4KO-ES cells. (A). Undifferentiated 5T4KO-ES cells forced to express 5T4 following infection with RAd-m5T4, (multiplicity of infection  = 30, dark grey columns) show no CXCL12 dependent chemotaxis comparable to undifferentiated WT-ES cells (black columns), mock (white columns), or RAd-eGFP (light grey columns) infection. (B), Differentiating 5T4KO ES cells with 5T4 expression restored by RAd-m5T4, (multiplicity of infection  = 30, dark grey columns) show CXCL12 chemotaxis comparable to differentiating WT-ES cells (black columns) but not following mock (white columns), or RAd-eGFP (light grey columns) infection. (C), Differentiating 5T4KO-ES cells chemotactic response to CXCL12 is also restored following infection with a recombinant adenovirus encoding human 5T4 (multiplicity of infection  = 30, black columns).

To examine biological response to CXCL12, WT and 5T4KO-ES cells were tested for CXCL12 chemotaxis before and after differentiation. Both WT and 5T4KO undifferentiated ES cells showed no chemotaxis towards CXCL12. Upon differentiation WT-ES cells showed a >2-fold response, while 5T4KO-ES cells remained unresponsive (Figure 1E). Following LIF withdrawal, both WT and 5T4KO-ES cells undergo an EMT with the tightly packed ES colonies becoming dispersed with the differentiating cells showing an arborized morphology. Although the differentiating 5T4KO-ES cells show reduced motility [16], their failure in chemotaxis was not a result of delayed kinetics in response since daily testing for up 6 days still provided no evidence for CXCL12 dependent chemotaxis (data not shown). The specificity of the chemotaxis was confirmed by showing that the differentiated WT-ES cell chemotaxis to CXCL12 was blocked by specific antibodies to the chemokine (Figure 1F) and by blocking the CXCR4 receptor with the inhibitor AMD3100 (Figure 1G). Further, the lack of chemotaxis of differentiating 5T4KO-ES cells was not the result of continued CD26 activity destroying CXCL12, since pre-incubation with the competitive CD26 inhibitor diprotin A did not restore chemotactic behavior (Figure 1H). To test whether 5T4 might play a role in CXCL12 dependent chemotaxis, undifferentiated and differentiating 5T4KO-ES cells were infected with recombinant adenoviral vector encoding mouse 5T4 (RAd-m5T4) or RAd-GFP control vector. There was no change in chemotaxis of either WT or 5T4KO undifferentiated ES cells infected with the different vectors (Figure 2A). Expression of m5T4 in differentiating 5T4KO-ES cells restores CXCL12 chemotaxis comparable to that of differentiating WT-ES cells (Figure 2B). A recombinant adenovirus encoding human 5T4 also restored chemotaxis (Figure 2C). These data suggest that 5T4 expression is a necessary cofactor for CXCR4 functional expression and CXCL12 chemotaxis in differentiating ES cells. One mechanism that might account for these results would be if 5T4 molecules facilitate stable cell membrane expression of CXCR4 molecules in differentiating ES cells.

### 5T4 expression influences plasma membrane expression of CXCR4 in differentiating ES cells

**Figure 3:**
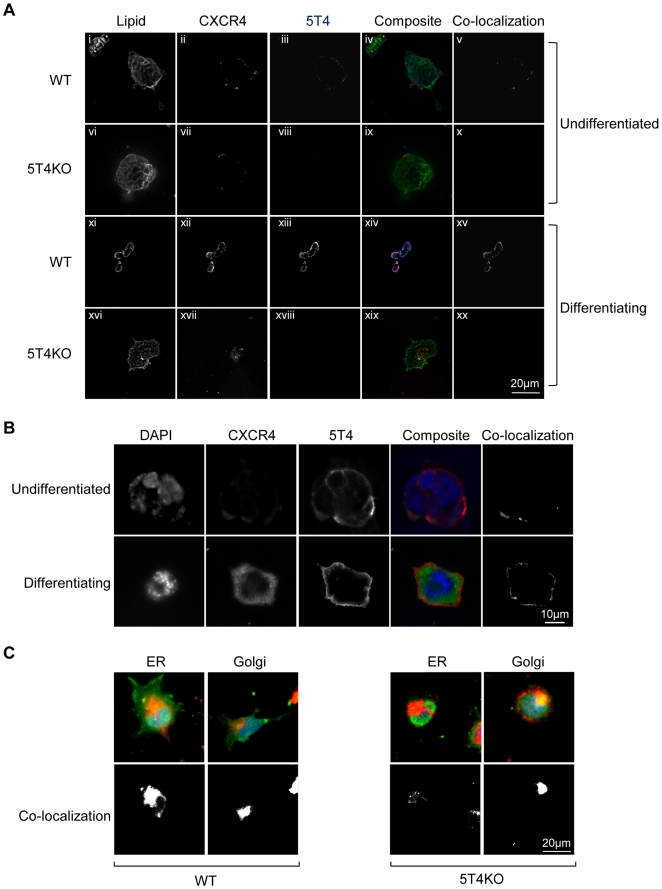
Cellular location of CXCR4, 5T4 in undifferentiated and differentiating WT and 5T4KO-ES cells. (A), Shows lipid rafts in the membrane of all cells (i, vi, xi, xvi); CXCR4 is intracellular in undifferentiated WT-ES and all 5T4KO-ES cells (ii, vii) and cell surface 5T4 is only expressed on differentiation of WT-ES cells (xiv). The composite images (lipid  =  green, CXCR4  =  red and 5T4  =  blue) show co-localization of 5T4 and CXCR4 (purple) including in lipid rafts (white) in differentiating WT-ES cells (xiv) but no other cells (iv, ix, xix) (co-localized areas are shown in separate channel (v, x, xv, xx)). (B), RAd-m5T4 infection of 5T4KO-ES cells leads to cell surface expression of both 5T4 and CXCR4 only in differentiating cells but not in undifferentiated cells which are seen to co-localize (CXCR4  =  green, 5T4  =  red) in the composite (yellow)(co-localized areas are shown in separate channel). RAd-GFP showed no effect on CXCR4 expression (not shown). (C), Upper panels, Double labeling of WT or 5T4KO-ES cells with either NBD C_6_-Ceramide (Golgi) or Endotracker (ER) (both red) shows that in the absence of 5T4, CXCR4 (green) accumulates predominately in the Golgi and to a lesser extent the smooth ER (yellow) whereas cell surface labeling is apparent only in the differentiating WT-ES cells)(lower panels: co-localized areas are shown in separate channel).

The expression and cellular localization of 5T4 and CXCR4 molecules before and after differentiation of WT and 5T4KO-ES cells was determined by immunofluorescence of fixed cells grown on glass plates (Figure 3A). Undifferentiated WT-ES cells are 5T4-negative with CXCR4 expression low and intracellular however following differentiation both molecules can be detected at the cell surface with clear areas of co-localization. By contrast, differentiated 5T4KO ES cells show only intracellular CXCR4 expression. Quantitatively, 98% of differentiating WT-ES cells showed cell surface CXCR4 expression and only 2% cytoplasmic while differentiating KO-ES had 1% cell surface and 89% cytoplasmic CXCR4 labeling; 10% were CXCR4 negative. It is apparent that at least some 5T4 and CXCR4 molecules co-localize to lipid rafts in differentiating WT but not 5T4KO differentiating ES cells where CXCR4 remains intracellular. However, when differentiating 5T4KO-ES cells are infected with RAd-m5T4, CXCR4 can be detected at the cell surface co-localized with 5T4 molecules (Figure 3B). RAd-m5T4 infected undifferentiated WT-ES cells show only limited CXCR4 and 5T4 surface expression in a few outer cells of undifferentiated ES colonies. These are most likely spontaneously differentiating cells suggesting that differentiation is a necessary cofactor for co-localization/expression of CXCR4 and 5T4 at the cell surface. In differentiating 5T4KO-ES cells, CXCR4 accumulated in the Golgi and to a lesser extent in smooth endoplasmic reticulum (Figure 3C). These data are consistent with 5T4 molecules being necessary for the surface expression of the CXCR4 receptor and chemotaxis to CXCL12 in differentiating ES cells. These properties were further explored using MEF derived from WT and 5T4KO mice.

### CXCR4 cell surface expression, CXCL12 mediated ERK signaling and chemotaxis are 5T4 dependent in mouse embryo fibroblasts

**Figure 4:**
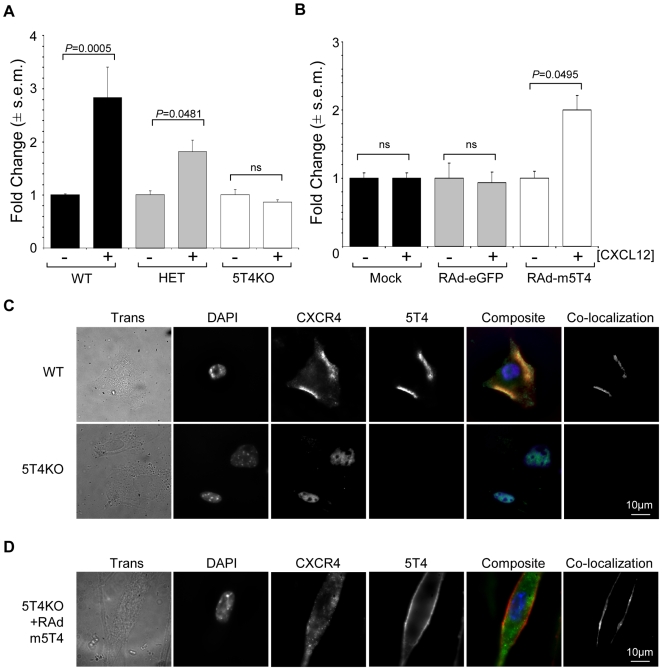
Role of 5T4 expression in the CXC12/CXCR4 axis in MEF. (A), MEF derived from wild-type, (WT, black columns), 5T4 heterozygote, (HET, grey columns) and 5T4 null, (5T4KO, white columns) embryos show 5T4 gene dose related CXCLl2 chemotaxis. (B), Chemotaxis of 5T4KO MEF following mock infection, (black columns), or infection with RAd-eGFP, (grey columns), or RAd-m5T4, (white columns); CXCL12 chemotaxis is only restored by RAd-m5T4. (+ or – 30 ng CXCL12). (C), Pattern of expression of CXCR4, (green) and 5T4, (red) in WT and 5T4KO MEF. In WT cells, CXCR4 and 5T4 are seen at the cell surface and clearly co-localize (CXCR4  =  green; 5T4  =  red; composite: co-localization  =  yellow; co-localized areas shown in separate channel) while in 5T4KO cells CXCR4 is located intracellularly around the nucleus; compare to DAPI labeling (blue). (D), 5T4KO MEF infected with RAd-m5T4 exhibit cell surface expression of both 5T4 and CXCR4 also displayed by co-localization (5T4 = red; CXCR4  = green; composite co localization =  yellow; co-localized areas shown in separate channel). RAd-GFP had no effect on CXCR4 expression (not shown).

A 5T4 dependency for CXCR4-mediated chemotaxis is also apparent in MEF as shown by: (1) a 5T4 gene dose influence on CXCL12 chemotaxis in WT, heterozygote and 5T4KO MEF (Figure 4A); (2) the restoration of the chemotactic response of 5T4KO MEF by RAd-m5T4 (Figure 4B); and (3) the co-localization of some CXCR4 molecules with typical 5T4 cell surface expression in WT MEF while 5T4KO MEF show only intracellular CXCR4 (Figure 4C) that can be rescued at the cell surface by RAd-m5T4 (Figure 4D). Detailed analysis of individual cells in this experiment documented 98% of WT MEF showing all CXCR4 at the cell surface. By contrast, 76% of 5T4KO MEF showed either CXCR4 perinuclear or cytoplasmic labeling and only 2% with any apparent membrane associated labeling; in 22% 5T4KO MEF CXCR4 was not detected.

**Figure 5:**
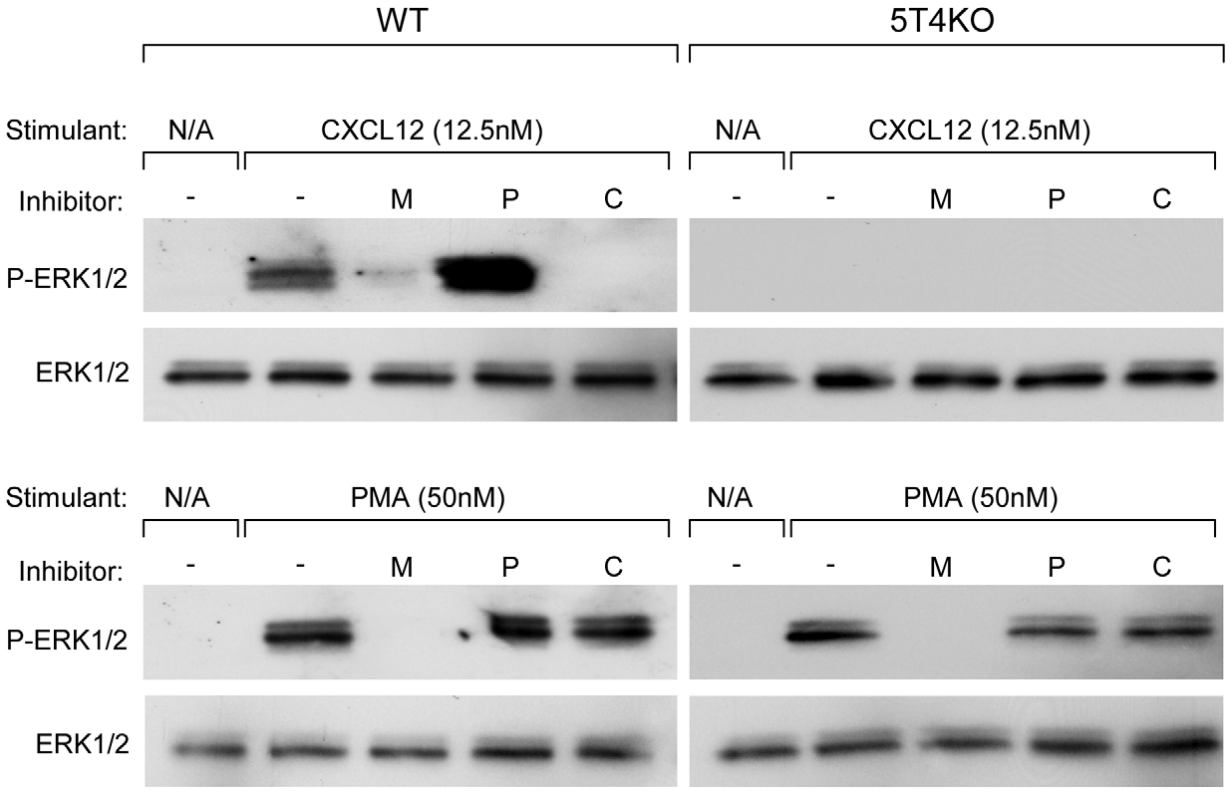
Disruption of cytoskeleton and CXCL12 dependent signaling in 5T4KO MEF. WT MEF exhibited an increase in ERK phosphorylation in response to CXCL12 stimulation that was prevented by the MEK1 inhibitor PD98059 (M, 50 µM) and the CXCR4 inhibitor AMD3100 (C, 10 µM) but not by the PI3K inhibitor LY294002 (P, 50 µM). 5T4KO MEF did not respond to CXCL12 stimulation, and this lack of response was specifically related to CXCR4 function and not due to a generalized disruption of MAPK/ERK signaling as both WT and KO MEF exhibited an increase in ERK1/2 phosphorylation in response to PMA stimulation, which was blocked by MEK1 inhibition but independent of both CXCR4 and PI3K activity. Total ERK was used as a loading control.

CXCL12 activates the MAPK/ERK signal transduction pathway. We therefore examined the requirement for 5T4 in the activation of this pathway in WT and 5T4KO MEF (Fig. 5). Stimulation of WT MEF with the chemokine CXCL12 induced the phosphorylation of the key intracellular effector ERK1/2. The activation of ERK1/2 by CXCL12 was prevented by using a specific inhibitor of either an upstream kinase MEK (PD98059; Figure 5, Lane M) or of CXCR4 (AMD3100; Figure 5, Lane M) but not by an inhibitor of PI3K (LY294002; Figure 5, Lane P) in a different signaling pathway, demonstrating that signal transduction was dependent upon CXCR4 mediated activation of the MAPK/ERK pathway. In 5T4KO MEF, CXCL12 activation did not lead to ERK1/2 phosphorylation, however this was not due to an overall disruption of the MAPK/ERK pathway as Phorbol 12-Myristate 13-Acetate, (PMA) stimulation led to a MEK but not CXCR4 dependent phosphorylation of ERK1/2 in both genotypes. These data demonstrate that in WT MEF a classical signal transduction pathway evoked by CXCL12 mediated activation of CXCR4 is functional. However, in the absence of 5T4, the chemokine receptor is no longer able to activate this pathway and the phosphorylation status of ERK1/2 is not responsive to CXCL12.

### The transmembrane domain of 5T4 is necessary for CXCR4 cell surface expression

**Figure 6:**
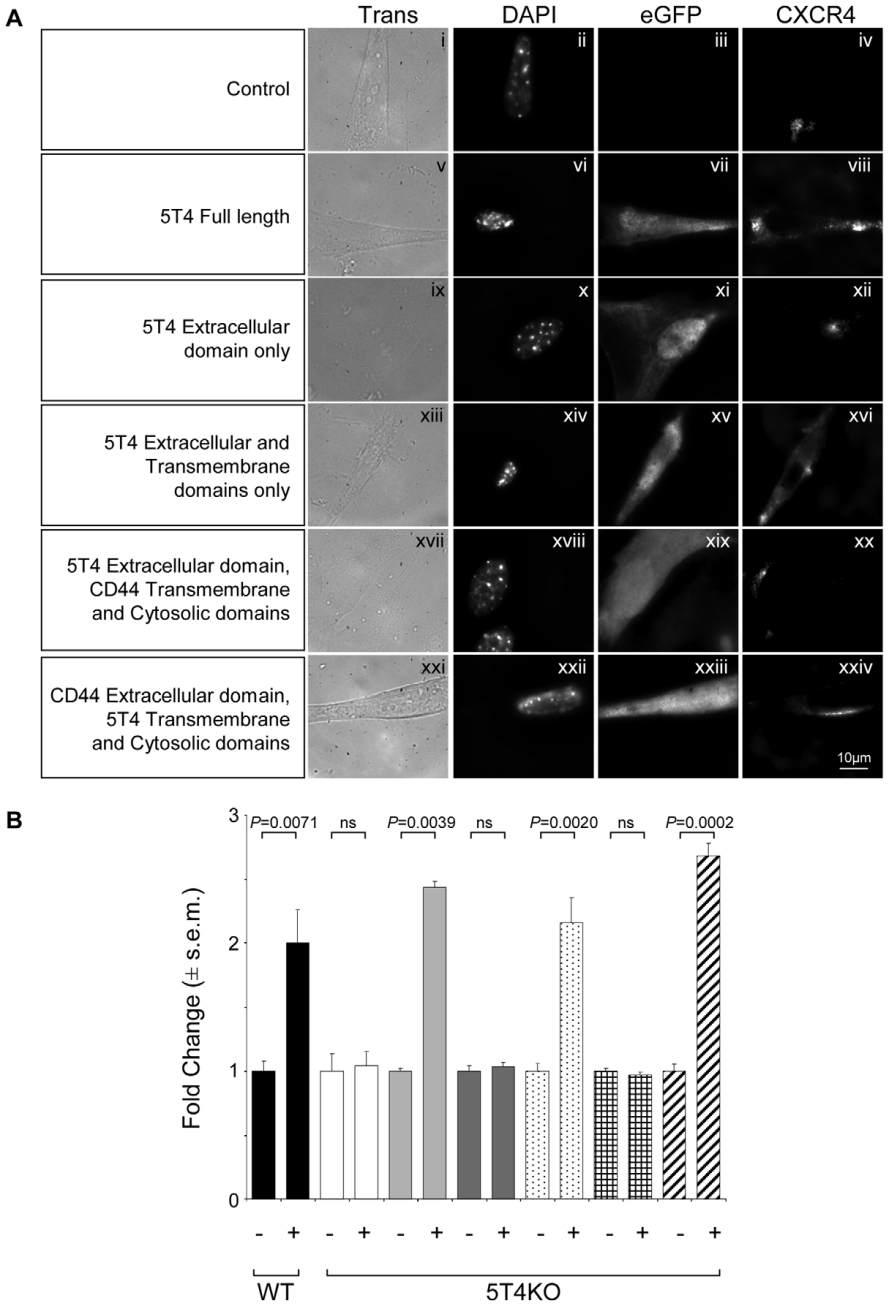
The transmembrane domain of 5T4 is necessary for CXCR4 cell surface expression. (A), 5T4KO MEF were transduced with retroviral vectors encoding both eGFP and full length or truncated 5T4 or chimeric 5T4/CD44 constructs. Successful infection was assessed by GFP expression and the location of CXCR4, assessed in these cells. Cell surface expression of CXCR4 is only seen with constructs containing 5T4 TM (viii, xvi, xxiv); the extracellular and cytoplasmic domains of 5T4 are not required. (B), Consistent with this CXCL12 chemotaxis of the retrovirally transduced GFP+ 5T4 null MEF with 5T4 extracellular domain, (dark grey), 5T4 extracellular domain CD44 transmembrane and cytosolic domains, (grid) and mock infected, (white) showing no affect whereas full length 5T4, (light grey), 5T4 extracellular and transmembrane domains, (spots) and CD44 extracellular domain 5T4 transmembrane and cytosolic domains, (stripes) show comparable levels to wild-type (black columns).

In the embryonic cells investigated it appears that cell surface expression of, and chemotactic response through, CXCR4 can be regulated by 5T4 expression. To examine the role of the extracellular, transmembrane and cytoplasmic domains of 5T4 molecules in CXCR4 surface expression, a series of murine 5T4 gene plasmid constructs were generated and cloned into a retrovirus also encoding eGFP as a reporter gene. 5T4KO MEF were infected with the retroviral constructs and cells were examined for both eGFP expression and CXCR4 localization by immunofluorescence (Figure 6). 5T4KO fibroblasts (controls, Figure 6A, panel i-iv) infected with retroviruses encoding full-length 5T4 (Figure 4a, panels v-viii) showed surface expression of CXCR4. However, the 5T4 extracellular domain was insufficient (Figure 6A, panels ix-xii) and the cytoplasmic domain unnecessary (Figure 6A, panels xiii-xvi) for CXCR4 expression on the cell surface. To test whether the 5T4 transmembrane domain (TM) was necessary and sufficient for cell surface CXCR4 expression, chimeric constructs of mouse 5T4/CD44 molecules with reciprocally exchanged TM and cytoplasmic domains were engineered. CD44 gene was selected for this experiment since, similarly to 5T4, it is a transmembrane glycoprotein, involved in adhesion and motility. It is constitutively expressed in MEF with no effect on surface CXCR4 expression. Importantly, cells infected with the retrovirus encoding the 5T4 extracellular domain fused to the transmembrane and cytoplasmic region of CD44 exhibited no cell surface expression of CXCR4 (Figure 6A, panels xvii-xx), whereas the reciprocal construct did promote cell surface expression of CXCR4 (Figure 6A, panels xxi-xxiv). Similar results were obtained with transfection of the plasmid constructs, confirming that these results were not an artifact of the viral infections (not shown). More importantly, when the biological function of these cells were assessed there was a clear correlation between the ability of the constructs to promote cell surface expression of CXCR4 and their ability to migrate towards CXCL12 (Figure 6B). Only constructs which contained the TM of 5T4 exhibited both cell surface expression of CXCR4 and CXCL12 mediated chemotaxis. Together these observations suggest that the transmembrane region of the 5T4 glycoprotein is required for the surface expression of CXCR4 in MEF and consequently their ability to respond to CXCL12 chemotactically.

### Effects of cytoskeleton, microtubule and Golgi disruption on the co-localization pattern of 5T4 and CXCR4

**Figure 7:**
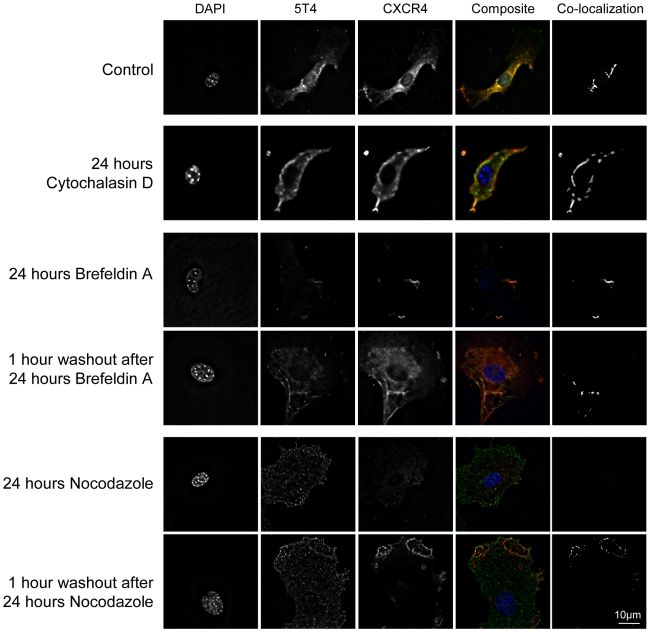
Effects of cytoskeleton, microtubule and Golgi disruption on the co-localisation pattern of 5T4 and CXCR4. Primary murine embryonic fibroblasts were assessed for their pattern of 5T4 and CXCR4 expression by immunofluorescence following 24 hours disruption of either the cytoskeleton (cytochalasin D), Golgi (brefeldin A) or microtubules (nocodazole) and 1 hour after drug washout. Cell surface expression of 5T4 (green) and CXCR4 (red) with regions of co-localization of the two antigens (seen as yellow) (also shown by co-localization analysis) are depicted.

To document the basic components of trafficking, primary WT MEF were treated for 24 hours with cytochalasin D, brefeldin A or nocodazole to disrupt the cytoskeleton, Golgi or microtubules respectively and the pattern of 5T4 and CXCR4 expression was determined before and after washout of the drugs. The cytoskeleton, Golgi and microtubule disruption was monitored by immunofluorescence with flurochrome conjugated phalloidin, NBD C6 ceramide and antibodies against β-tubulin respectively (not shown). Untreated primary MEF exhibit cell surface expression of both 5T4 and CXCR4 with considerable co-localization (Figure 7). After cytochalasin D treatment, there was no reduction in the CXCR4 cell surface expression or co-localization of 5T4 and possibly an increase compared to untreated controls. Brefeldin A reduced levels of cell surface expression of both antigens and all residual CXCR4 or 5T4 labeling was co-localized at cell surface. One hour after brefeldin A washout, increased cell surface expression of both antigens with marked cell surface co-localization was observed. Following nocodazole treatment there was some intracellular accumulation but no cell surface detection of CXCR4. 5T4 remained detectable at the cell surface albeit at a diminished level and no co-localization with CXCR4 was visible. One hour after nocodazole washout both antigens were detectable at the cell surface with marked co-localization. Clearly, detection of plasma membrane co-localized 5T4/CXCR4 molecules is dependent on microtubules and the molecules are not obligatorily associated at the Golgi. Disruption of the Golgi or the actin cytoskeleton per se does not disrupt all 5T4/CXCR4 co-localization at the plasma membrane. It appears that CXCR4 and 5T4 molecules can form a stable interaction at the cell surface facilitating the biological response to CXCL12.

### Inhibition of CXCL12 chemotaxis by monoclonal antibodies recognizing m5T4 in mouse embryonic cells

**Figure 8:**
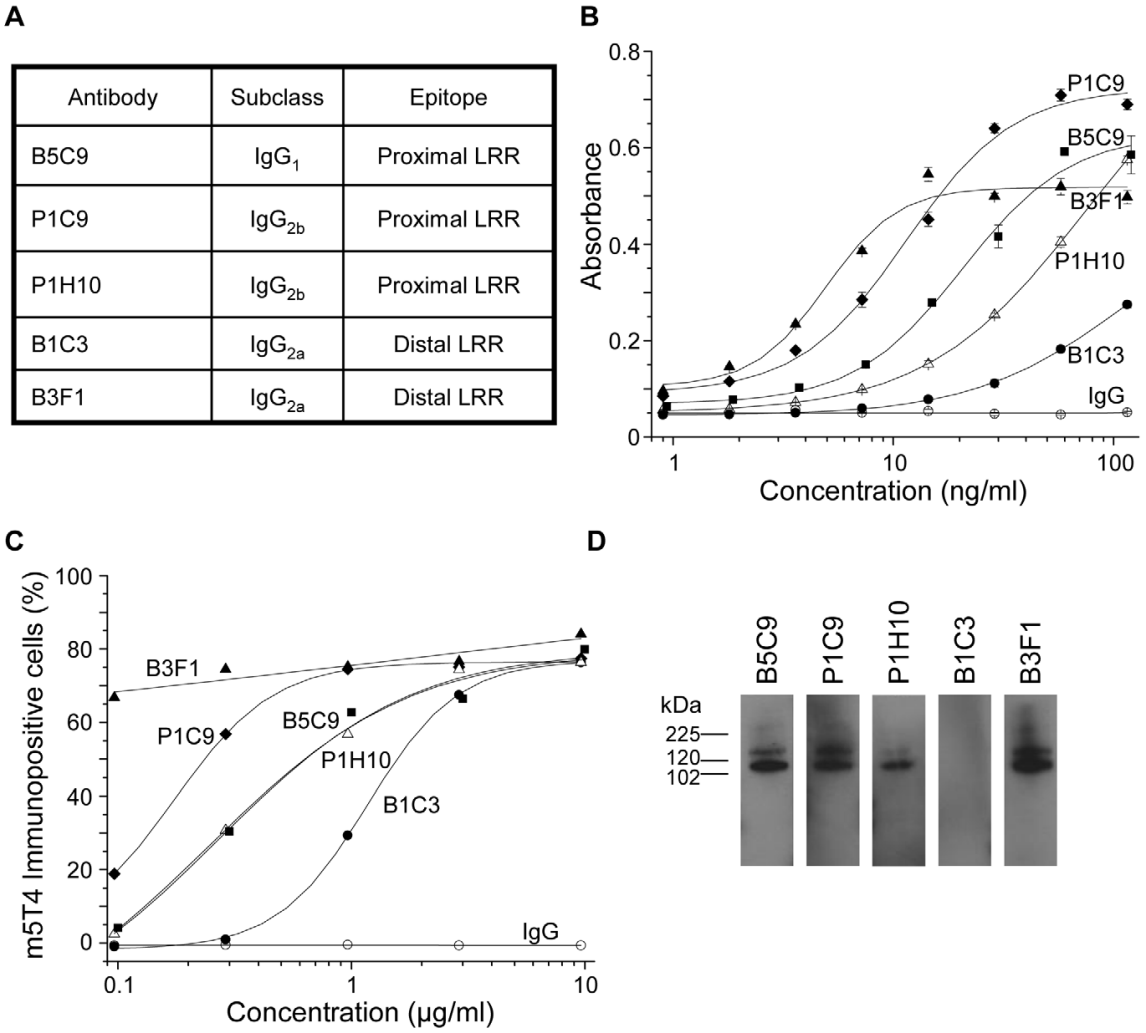
Characterization of m5T4 specific mAbs. (A), Summarizes the IgG subclasses of five m5T4 specific monoclonal antibodies (mAb, made in 5T4KO mice) recognizing distinct epitopes in the proximal and distal 5T4 extracellular LRR containing domains. (B), Shows titration of mAb activity in m5T4 specific ELISA [27]. (C), Shows titration of mAb cell surface labeling of B16m5T4 tumor cells by flow cytometry. (D), Western blot analysis of mAb probing against recombinant m5T4-pIg showing recognition of m5T4 by all mAbs except B1C3.

**Figure 9:**
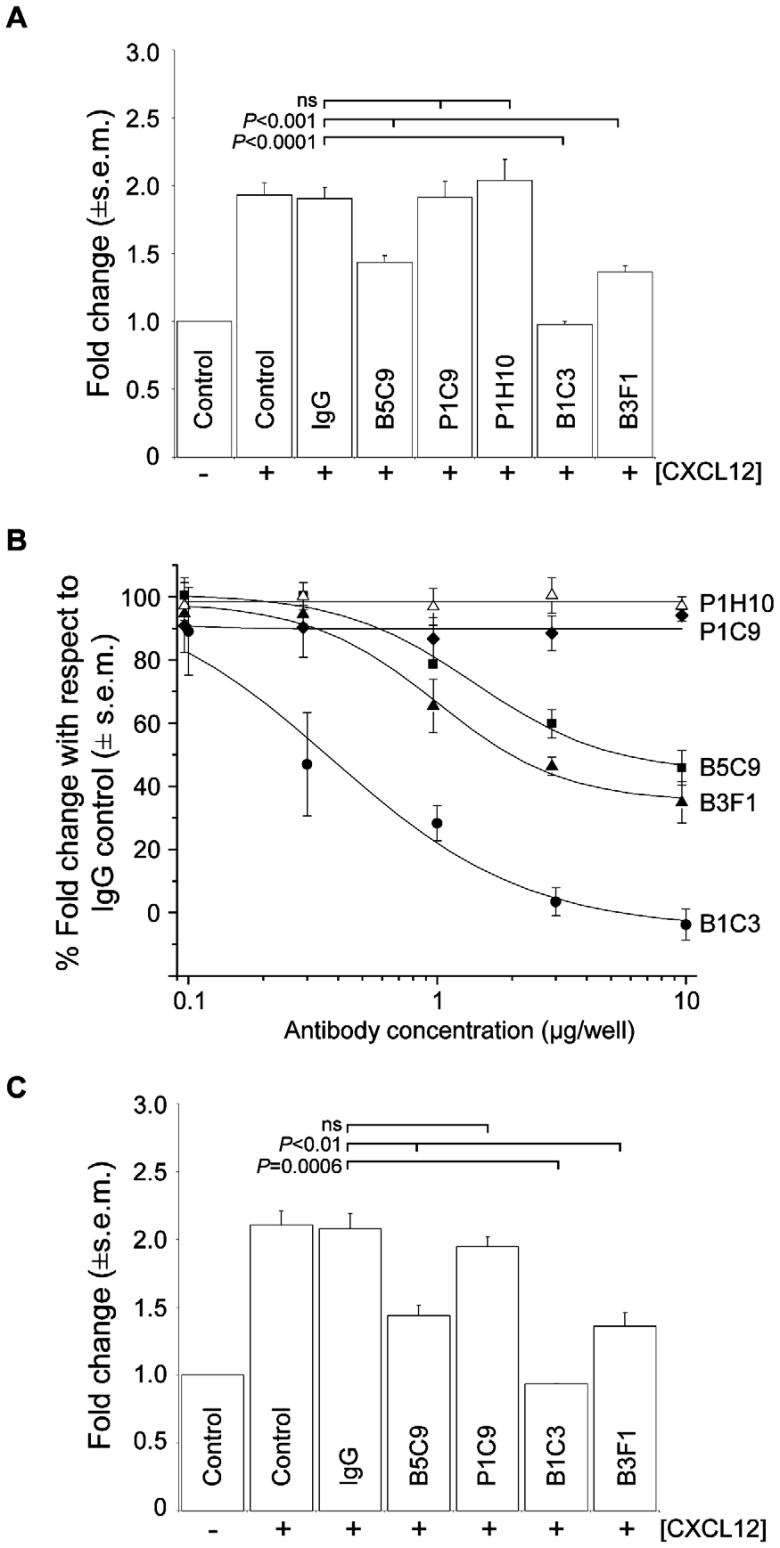
Inhibition of chemotaxis by monoclonal antibodies recognizing 5T4. (A), The chemotactic migration exhibited by differentiating WT-ES cells towards CXCL12 was abolished in the presence of the m5T4 specific mAb B1C3 (10 µg) but not in presence of mAb P1C9 or P1H10 (10 µg) or an irrelevant control antibody (10 µg). MAbs B3F1 and B5C9 (10 µg) reduced the chemotactic response. (−  =  no CXCL12, +  =  10ng CXCL12). (B), MAb dose response of inhibition of chemotaxis towards CXCL12 in differentiating WT-ES cells. (C), The chemotactic migration exhibited by primary WT MEF was abolished in the presence of the m5T4 specific mAb B1C3 (10 µg) but not in presence of mAb P1C9 (10 µg) or an irrelevant control antibody (10 µg). MAbs B3F1 and B5C9 (10 µg) reduced the chemotactic response.

5T4 molecules play a role in stabilizing plasma membrane expression of CXCR4 receptors most likely through interaction of their transmembrane domains. It is possible that the binding of antibodies recognizing the extracellular domains of 5T4 molecules might influence 5T4-CXCR4 interactions through modulation of cell surface expression or altering conformation. We investigated the ability of several different monoclonal antibodies to m5T4 to influence the chemotactic response of differentiating ES cells or MEF to CXCL12. Five different monoclonal antibodies recognizing distinct epitopes in the proximal and distal LRR domains of m5T4 were available (Figure 8A). Each antibody showed different affinity in a m5T4 specific ELISA (Figure 8B) or B16m5T4 FACS titration (Figure 8C), decreasing in the order B3F1, P1C9, B5C9, P1H10 and B1C3. All detect the extracellular domain of m5T4-Fc by western blotting except for B1C3 (Figure 8D). Importantly, the chemotactic migration towards CXCL12 exhibited by differentiating WT-ES cells was abolished in the presence of mAb B1C3 but not P1C9 or P1H10 while B3F1 and B5C9 showed less but still significant inhibition of the chemotactic response (Figure 9A). Figure 9B shows titration of antibody inhibition of differentiating ES cell chemotaxis for the mAbs; the IC_50_ for the maximally inhibitory mAb B1C3 was 0.36 µg, ±0.11 and for B3F1 and B5C9 was 2.2 µg±0.8 and 6.8 µg±1.5 respectively. The inhibition of chemotaxis by the mAb B1C3 was prevented by the presence of m5T4-Fc (not shown). Similar results were exhibited by primary WT MEF for four of the monoclonal antibodies tested (Figure 9C). Thus, the chemotactic response of both differentiated ES cells and MEF can be blocked by some but not all antibodies recognizing distinct parts or epitopes of m5T4 molecules.

The possibility that the inhibitory antibodies might differentially modulate cell surface 5T4 expression was tested. Flow cytometry analysis showed no significant differences in the modulation of cell surface expression of 5T4 molecules after 2 or 24 hours by P1C9 and B1C3 mAbs as assessed in B16m5T4 cells (data not shown). To assess any differential influence on recycling of CXCR4 molecules at the cell surface, MEF were treated with nocodazole to remove CXCR4 membrane expression and allowed to recover in the presence of either P1C9 or B1C3 mAbs. There was no significant difference in recovery of CXCR4 membrane expression in the presence of non-inhibitory P1C9 or inhibitory B1C3 mAb after 3 or 6 hours (data not shown). These data suggest that the influence of inhibitory 5T4 antibodies is not mediated through preventing colocalization of 5T4 and CXCR4 at the cell surface.

### 5T4 facilitates CXCR6 but not CXCR3 or CXCR7 cell surface expression and response to specific ligands

While the sequence variation across the extracellular chemokine receptor domains may provide for ligand specificity, mechanisms of transmembrane domain interaction with 5T4 molecules may be shared with other molecules in the CXC receptor family. Since, in addition to CXCL12 and CD26, the microarray data identified significant upregulation of transcript levels in ES differentiation of the chemokine CXCL10, and because CD26 is able to regulate multiple chemokines, a role for 5T4 in the expression and function of other chemokine receptors, including the CXCL10 receptor CXCR3 [34] and the other CXCL12 receptor CXCR7, was investigated.

**Figure 10:**
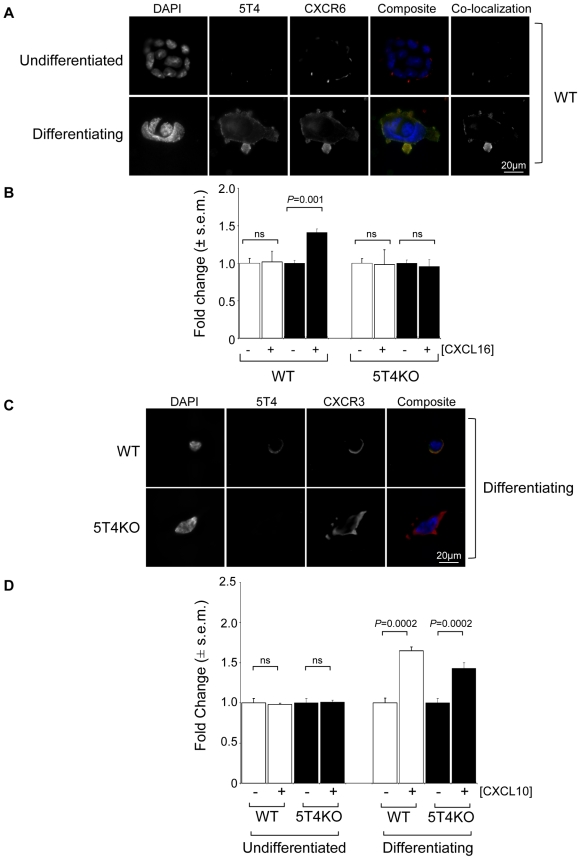
Influence of 5T4 on the chemokine receptors CXCR6 and CXCR3 in ES cells. (A), The expression and cellular localization of 5T4 and CXCR6 molecules on undifferenetiated and differentiating WT-ES cells was determined. Both molecules can be detected at the cell surface in differentiating cells with some areas of co-localization (5T4  =  green; CXCR6  =  red; composite, co-localisation  =  yellow; co-localized areas shown in separate channel). (B), Undifferentiated (white columns) and differentiating (black columns) ES cells were placed in a gradient of chemokine CXCL16 or not. (C), Immunofluorescence detection of 5T4 and CXCR3 in differentiating WT and 5T4KO-ES cells (5T4  =  green, CXCR4  =  red). Cell surface expression of 5T4 is present only on differentiating WT-ES cells whilst cell surface expression of CXCR3 is evident in both undifferentiated and differentiating WT and KO ES cells. (D), Undifferentiated WT (white columns) and 5T4KO (black columns) ES cells exhibit no CXCL10 dependent chemotaxis but differentiating WT and 5T4KO-ES cells, acquire significant chemotaxis towards CXCL10.

The expression and cellular localization of 5T4 and CXCR6 molecules before and after differentiation was determined by immunofluorescence of fixed ES cells grown on glass plates. Undifferentiated WT-ES cells are 5T4-negative with CXCR6 expression low and intracellular. Following differentiation both molecules can be detected at the cell surface with some areas of co-localization (Figure 10A). To examine whether cell surface expression of CXCR6 was biologically functional, undifferentiated and differentiating WT and 5T4KO-ES cells were placed on a chemotactic gradient towards the chemokine CXCL16, which is a known ligand for CXCR6. Undifferentiated ES cells of either genotype exhibited no chemotaxis towards CXCL16. In contrast, differentiating WT-ES cells exhibited a significant increase in chemotaxis towards CXCL16 whilst differentiating 5T4KO-ES cells showed no functional chemotaxis towards CXCL16 (Figure 10B). This phenomenon was also evident in WT MEF with co-localization of 5T4 and CXCR6 on the cell surface. By contrast, 5T4KO MEF showed no cell surface expression of CXCR6. Analysis of cell surface expression of CXCR6 by flow cytometry confirmed results obtained by immunocytochemistry, showing CXCR6 cell surface expression on WT but not 5T4KO MEF (data not shown). Thus CXCR6 expression and its response to the chemokine CXCL16 does require 5T4 expression in embryonic mouse cells.

In contrast to CXCR4 and CXCR6, CXCR3 expression and its response to its ligand CXCL10 does not require 5T4 expression. Expression of CXCR3 is found at the cell surface of both WT and 5T4KO undifferentiated (not shown) and differentiating ES cells (Figure 10C). However, a chemotactic response to CXCL10 is only seen after differentiation in both WT and 5T4KO-ES cells (Figure 10D). Further analysis of undifferentiated ES cells treated with the inhibitor Diprotin A (10 µM) suggests that lack of response in undifferentiated ES cells is partly due to CD26 activity which can destroy CXCL10 [35] (data not shown). Treatment of both WT and 5T4KO differentiating ES cells with the G_i_ protein inhibitor pertussis toxin (10 ng/ml) showed that G_i_ protein-chemokine receptor interaction occurs irrespective of 5T4 expression at the cell surface allowing CXCL10 chemotaxis (data not shown).

**Figure 11:**
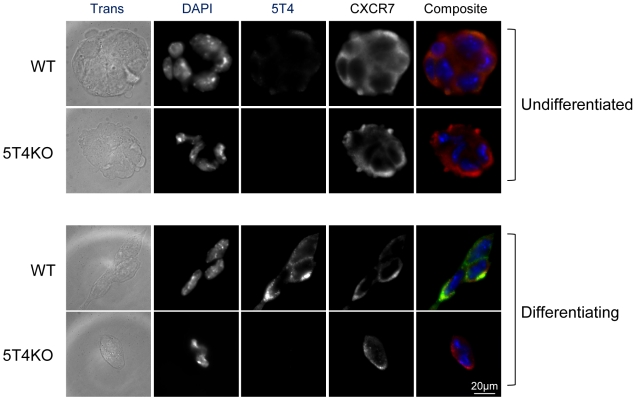
5T4 is not required for CXCR7 surface expression. Immunofluorescence detection of 5T4 and CXCR7 in undifferentiated and differentiating WT and 5T4KO-ES cells (5T4  =  green, CXCR7  =  red). Cell surface expression of CXCR7 is high in undifferentiated ES cells (either WT or 5T4KO). In 3 day differentiating WT-ES cells, CXCR7 is relatively downregulated from the cell surface whereas in differentiating 5T4KO-ES surface CXCR7 is retained.

Similarly cell surface expression of CXCR7 is also independent of 5T4 cell surface expression. Undifferentiated ES cells (either WT or 5T4KO) demonstrated both cytoplasmic and cell surface expression of CXCR7 (Figure 11). In contrast following 3 days under differentiating condition in WT-ES cells, CXCR7 is relatively down-regulated from the cell surface whereas in differentiating 5T4KO-ES surface CXCR7 is retained (Figure 11).

## Discussion

In this study, we have demonstrated that in mouse differentiating ES cells and embryonic fibroblasts, 5T4 glycoprotein plays a critical role in the membrane expression of CXCR4 and the chemotactic response to CXCL12. We first showed that during ES cell differentiation, increased surface expression of 5T4 and CXCL12 production was accompanied by decreased membrane expression of the CXCL12 regulatory protease CD26 whereas total cell levels of CXCR4 were unchanged. Studies with 5T4KO and WT-ES cells and MEF established that 5T4 molecules are required for cell surface expression and intracellular signaling of the CXCL12 receptor CXCR4. Importantly, chemotaxis in response to CXCL12 is disrupted in the absence of 5T4. The 5T4 dependency for CXCR4 plasma membrane expression and co-localization studies support the direct molecular interaction of CXCR4 and 5T4 molecules. The transmembrane region of 5T4 is critical for this purpose as it was sufficient in the context of CD44 molecules to allow functional surface CXCR4 expression and chemotaxis after introduction to 5T4KO MEF. Although dependent on the TM of the 5T4 molecule, the precise nature and stability of the interaction has not yet been ascertained. There is evidence that CXCR4 molecules form homo- and heterodimers [36] and LRR domains of the 5T4 molecules provide the biochemical basis for formation of multimers [37] so the stoichiometry is complicated to predict. It is possible that lipid rafts and/or additional molecules may also be a component part of a “functional” complex. The lifespan of the primary MEF have limited availability for biochemical studies. Attempts to detect a physical complex of 5T4 and CXCR4 have been successful in human tumor cells which exhibit higher levels of 5T4 expression and where pulldown and reciprocal western analyses suggest association of some 5T4 and CXCR4 molecules in non-ionic detergent solubilized lysates (Southgate et al unpublished). These observations need further study using cross-linked membrane preparations as well as FRET studies.

In order to distinguish where the CXCR4 and 5T4 interaction might occur, we studied the effects of cytoskeleton, microtubule and Golgi disruption on the co-localization pattern of the two molecules in MEF. CXCR4 intracellular trafficking to the cell surface post Golgi is dependent on the microtubules and not the actin cytoskeleton. Once at the plasma membrane, co-localized CXCR4 and 5T4 molecules appear to show a stable association. There was some indication that cytochalasin increased CXCR4/5T4 expression at the cell surface. The observation that following actin depolarization 5T4 and CXCR4 remain at the plasma membrane is consistent with several reports implicating actin binding proteins in CXCR4 internalization and degradation[38]. For example, actin binding cortactin and plectrin have been shown to be involved in CXCL12 induced CXCR4 internalization and recycling in HEK293 cells overexpressing CXCR4 [39], [40]. Also, myosin IIA, a molecular motor involved in vesicle and protein trafficking along actin filaments, requires its actin binding domain for efficient endocytosis of CXCR4 [41]. Therefore the disruption of the cytoskeleton may have diminished the ability of 5T4 and CXCR4 to be endocytosed and subsequently degraded in the lysosomes thereby increasing their stability and half-life at the cell surface.

**Figure 12:**
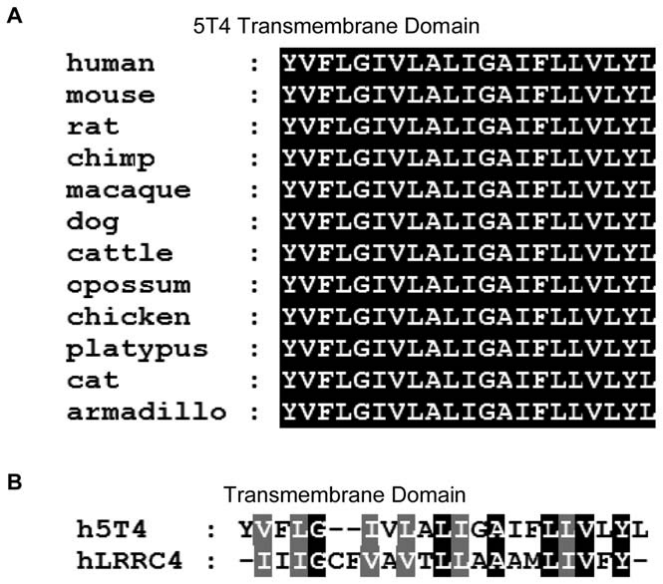
Sequence comparison of 5T4 TM domains. Alignments were performed using the ClustalW2 multiple sequence alignment program (EMBL-EBI) to compare (A), the TM domains of 5T4 across species and (B), the TM domain of human 5T4 with human LRRC4, identical residues shaded black, similar residues shaded grey.

The 5T4 gene is highly conserved across different vertebrate species and the TM region is completely conserved (Figure 12A). This explains why the human 5T4 and mouse 5T4 genes could equally restore CXCR4 cell surface expression in 5T4KO cells. Chemokine receptors, G-protein-coupled seven TM spanning proteins, are also highly conserved in evolution [42], with the hydrophobic amino acids of TM domains forming α-helical structures which anchor the receptors in the membrane [43]. Current understanding of the mechanisms of CXCL12-CXCR4 interaction support a two step mechanism where by the initial interaction occurs between the ligand β-sheet and its 50S and N-loop and the CXCR4 extracellular region which facilitates rapid binding and efficient anchoring on the extracellular side of the receptor [44]. The ligand N-terminus remains highly dynamic and searches for the binding cavities buried with the receptor TM helices. This second step interaction between the ligand N-terminus and the receptor TM region triggers conformational changes in the CXCR4 TM to induce G-protein signaling [44]. Importantly, the chemotactic response of both differentiated ES cells and MEF can be blocked by some but not all antibodies recognizing distinct parts and epitopes of m5T4 molecules. These data suggest that 5T4 contributes to functional integrity of the CXCR4 receptor expression at the cell surface. 5T4 antibody induced modulation of 5T4 molecules from the cell surface or prevention of CXCR4/5T4 co-localization do not appear to account for observed inhibition of some m5T4 mAbs. It seems more likely that the inhibition results from allosteric effects on CXCR4 altering the nature of ligand binding or its consequences. Since chimeric CD44/5T4TM molecules are functional in facilitating CXCR4 membrane expression and chemotactic response one might conclude that the 5T4 specific antibodies are unlikely to influence the initial direct binding of CXCL12. The most efficient inhibitory antibody (B1C3) showed differential activity in ELISA and western blotting of m5T4-Fc compared to the other mAbs, consistent with an epitope influenced partly by the presence of the 5T4 TM domain. It is possible that the 5T4 transmembrane region specifically recognizes intramembrane residues of CXCR4 and contributes not only to the stability of the CXCR4 expression in the plasma membrane but possibly also to conformational changes in the receptor which govern responsiveness to ligand. The latter may be influenced by binding of antibodies to sequences in both the proximal and distal domains of the 5T4 molecules. It is also conceivable that the 5T4-CXCR4 interaction may also influence receptor fate by modulating internalization events controlled by β-arrestin binding to the activated receptor or of targeting for degradation by preventing ubiquination and trafficking through early endosomes to the lysosome or by providing opportunity for recycling to the cell surface through the 5T4 PDZ motif which is a mechanism used by some other G protein binding receptors [27]. Indeed, 5T4 molecules also influence aspects of cytoskeletal organization including through the cytoplasmic domain [3], [4], [5] and these may be an integrated component of chemotactic response/motility. These possibilities require further investigation.

We have shown that in differentiating ES cells and MEF, CXCR6 response to CXCL16 is also 5T4 dependent. However, CXCR3 and its response to CXCL10 is not 5T4 dependent and CXCR7 is constitutively expressed at the cell surface in undifferentiated ES cells. Interestingly, the LRR-containing protein LRRC4 has been reported to regulate both the expression and signal conduction of the CXCR4 receptor. Introduction of LRRC4 into glioblastoma cells reduced CXCR4 expression, CXCL12-induced ERK and AKT phosphorylation and matrix metalloproteinase expression [45]. Crucially, the TM regions of 5T4 and LRRC4 are similar but contain significant differences (Figure 12B). Absence of another membrane protein, Robo1, or of its ligand Slit was also shown to up-regulate the CXCL12/CXCR4 signaling in mammary epithelium [46]. Robo 1 has a distinct TM domain from 5T4. All these observations point to multiple and complex control of CXCL12/CXCR4 signaling which may have developmental stage- or tissue-specific elements. The delineation of embryonic cells with distinct chemotactic responses and where 5T4 expression is one of the controlling factors is consistent with early differentiation of ES cells being representative of events around implantation of the embryo at which point 5T4 expression is first detected [47]. The role of auxiliary proteins in receptor expression and function is not unprecedented. For example certain G protein-coupled receptors associate with receptor activity modifying proteins (RAMPs) which are required for receptor trafficking, ligand binding and receptor specificity [48].

It is clear that not all CXCL12/CXCR4 responsive cells express 5T4 molecules. 5T4 cannot be an absolute requirement for CXCR4 activation because 5T4 knockout mice are viable, whereas both CXCL12 and CXCR4 KOs are lethal [49], [50]. Clearly, there must be some redundancy and other molecules must be able to regulate CXCR4 trafficking to and/or retention at the cell surface. Additionally, there is mounting evidence that chemokine receptors are able to form discrete functional units via heterodimerisation with other G-protein coupled receptors. In the case of CXCR4, heterodimerisation with the chemokine receptor CXCR7, which binds the same ligand CXCL12, can alter both the kinetics and the dynamics of CXCR4 responsiveness to CXCL12 [51].

CXCL12 is a homeostatic chemokine that, unlike other ELR- CXC chemokines, is angiogenic. CXCL12 binds to the widely expressed CXCR4 (exclusively) and the more restricted CXCR7 (which also binds CXCL11) [26]. CXCL12 through CXCR4 regulates cardiac and neuronal development, stem cell motility, neovascularisation and tumorigenesis [23]. Besides acting as a cofactor for HIV, CXCR4 mediates the CXCL12-directed migration of cancer cells to metastatic sites through the promotion of angiogenesis and migration of tumor cells in breast, lung, ovarian, renal, prostate and neuroblastoma [22], [23], [24]. It is significant that all these tumor types are known to express the 5T4 glycoprotein [2], [9], [52]. Importantly, these CXCR4-positive tumors preferentially spread to tissues with high levels of CXCL12 such as lung, liver, lymph nodes, brain and bone marrow which are key metastatic sites [22], [23], [24]. In addition, the stromal environment (often 5T4 positive [2], [7], [8]) can have a tumor-imprinted promotional influence [53]; and chemokines can sometimes induce proliferation rather than chemotaxis enhancing tumorigenesis [26], [54]. We have investigated the relationship between expression of 5T4, CXCR4 and chemotaxis in several human tumor cell lines including choriocarcinoma, breast and ovarian and all exhibited CXCL12-mediated chemotaxis and showed evidence of a 5T4/CXCR4 complex in the cell membranes (Southgate *et al*., unpublished). The regulation of CXCR4 surface expression by 5T4 molecules may provide a new way to control response to the chemokine CXCL12 in normal circumstances but could be selected to advantage the spread of a tumor from its primary site. If the latter events are preferentially and constitutively expressed properties of tumors then targeting the CXCR4/5T4 complex might offer new opportunities for therapeutic intervention.

## Materials and Methods

### Ethics Statement

All animal work was performed in accordance with the UK Animal Scientific Procedures Act 1986 and was covered by both Project and Personal licences that were issued by the Home Office and reviewed by the Paterson Institute for Cancer Research ethical committee. Professor Peter L Stern Home Office Project Licence numbers 40-2666 (years 2003–2008) and 40/3200 (years 2008–2013) and Named Animal Care & Welfare Officer Licence number 40/3085 covered all procedures and breeding. Local Ethics Committee approval was provided prior to submission of all subsequently approved project licence applications. Ethical Review Process advises the Certificate Holder regarding Project licence applications and standards of animal care and welfare; they also develop initiatives leading to the widest possible application of the 3Rs so that procedures are refined to minimise suffering, numbers of animals used are reduced and animal use is replaced wherever possible. Mice are housed in individually ventilated cages. These cages prevent the spread of potential disease from one cage to another and each cage has an individual Hepa filtered air supply that gives approximately 72 air changes per hour and a fixed exhaust system. All the cages are provided with environmental enrichment, in the form of nesting material, a variety of mouse houses, wooden chew blocks or play tunnels. The addition of these items increases socialisation and environmental stimulation for the mice and reduces aggression amongst some strains of males. Routine health screening from our colonies is performed to ensure that the mice are free from a list of specific pathogens (SPF) and any new strains brought into the unit are health screened before introduction into the facility.

### 5T4 Knockout (KO) mice

We have constructed a 5T4KO mouse by replacing the second exon of 5T4, which encodes the entire protein, with an IRES-LacZneo reporter gene in ES cells. These cells were used to produce chimeric mice and germline progeny; 5T4 KO heterozygote mice were backcrossed to the C57BL/6 background. The 5T4KO C57BL/6 animals are viable but adult animals show some structural disorganization within the brain and exhibit a high frequency of hydrocephalus. The frequency of hydrocephalus is approximately 13%, with the median age of death, (animals requiring termination) at 49 days, (range 38–83). We have made and characterized 5T4KO ES cells in order to study aspects of the role of 5T4 in EMT [16]. The 5T4KO mice were used to generate monoclonal antibodies specific for m5T4 (B3F1 (IgG_2a_); B5C9 (IgG_1_); B1C3 (IgG_2a_); P1C9 (IgG_2b_) and P1H10 (IgG_2b_). Primary murine embryonic fibroblasts (MEF) of all three genotypes were prepared from day 13 embryos following mating of male and female 5T4 heterozygote C57BL/6 transgenic mice by methods previously described [55].

### Cell lines

E14TG2a [56], (here referred to as WT-ES) and 5T4KO-ES cells [16] were cultured on pre-prepared 0.1% gelatine (Sigma) coated tissue culture flasks. ES cells were grown in Knockout DMEM, (Invitrogen) supplemented with 10% Hyclone fetal calf serum, (Perbio), 2 mM L-glutamine, 1% non-essential amino acids, (Sigma), nucleosides [6 ml of the following solution/500 ml DMEM: adenosine, (80 mg), guanosine, (85 mg), cytidine, (73 mg), uridine, (73 mg) and thymidine, (24 mg) dissolved in 100 ml double distilled water; Sigma], 2-mercaptoethanol, (50 µM; Invitrogen), leukemia inhibitory factor, (LIF; 1000 units/ml of ESGRO; Chemicon Int.), 100 units/ml penicillin and 100 µg/ml streptomycin (Gibco). For differentiation cells were grown in media that was not supplemented with LIF. Media was changed daily. MEF, A9 fibroblast cell lines, B16neo and B16m5T4 melanoma cell lines [57], [58] were cultured in DMEM supplemented with 10% fetal calf serum, 2 mM L-glutamine, 100 units/ml penicillin and 100 µg/ml streptomycin.

The hybridoma cell lines producing the anti-m5T4 monoclonal antibodies B5C9, P1H10, P1C9, B1C3 and B3F1 were cultured in DMEM supplemented with heat inactivated 10% fetal calf serum (FCS; Biosera), 2 mM L-glutamine, 100 units/ml penicillin and 100 µg/ml streptomycin solution and maintained at 37°C in a humidified atmosphere of 5% CO_2_/95% air. The hybridoma supernatant was clarified by centrifugation and proteins concentrated by precipitation with 45% saturated ammonium sulphate, dialysed extensively against PBS and antibodies purified on protein G chromatography (HiTrap protein G column, GE Lifesciences; [58]). The antibodies eluted with 100 mM glycine (pH 2.5) into 1 M Tris salt (pH 9) were dialysed against PBS and used for subsequent assays.

### Characterization of m5T4 specific monoclonal antibodies

Monoclonal antibodies were epitope mapped by FACS analysis performed against A9 cell lines bearing variant constructs of 5T4; m5T4, m/h5T4 or h/m5T4 chimeric constructs [59]. These cell lines were transfected with pCMVα neo constructs bearing either the full length m5T4, LRR1 of murine 5T4 fused to LRR2 of human 5T4 or LRR1 of human 5T4 fused to LRR2 of murine 5T4 respectively and maintained with 1mg/ml G418 selection (Sigma). Cells were suspended in FACS buffer (PBS, 0.2% bovine serum albumin, 0.1% sodium azide) and labelled with the appropriate concentration of anti-m5T4 monoclonal antibodies diluted in FACS buffer for 30 mins on ice, washed with FACS buffer and labelled with rabbit anti-mouse IgG conjugated to FITC (Dako, 1∶40). 10,000 events were acquired using a Becton Dickinson FACScan and the data obtained was analysed using WinMIDI (version 2.8) software.

Anti-m5T4 monoclonal antibodies were titrated by doubling dilution between 0 and 0.1 µg/ml by sandwich ELISA. 96-well ELISA plates (Falcon) were pre-coated at 4°C overnight with 1 µg/ml m5T4-pIgFc [58] in borate buffer (100 mM boric acid 150 mM NaCl; pH 8.5). All subsequent steps were performed at 37°C for 1 hour and the plates were washed three times with PBS containing 0.05% Tween 20 (Sigma; PBST) between each step. Non-specific binding was blocked using 2% low fat dried milk (marvel) in PBST (blocking buffer) at 37°C for 2 hours. Antibody binding was detected using goat anti-mouse IgG conjugated to HRP (Sigma, 1∶1000) in blocking buffer. The plates were developed using tetramethyl benzidine (TMB; Sigma), the colour reaction was stopped with 1 M sulphuric acid and the absorbance read at wavelength 450–650 nM. The isotypes of the monoclonal antibodies were determined using Isotyping kit (ISO-2; Sigma) according to the manufacture's instructions.

### Microarray analysis

We utilized the associated loss of pluripotency of murine ES cells with the early upregulation of 5T4 expression to search for other changes in gene expression using an Affymetrix approach [18]. The ES cells were grown with or without LIF for 3 days and the disaggregated cells sorted for expression of cell surface 5T4. E14 TG2a cells were investigated with samples showing minimal intra-replicate variance. Data were preprocessed using RMA[60], as implemented in the ‘affy’ BioConductor library[61] and then analysed using LIMMA [62] to identify those probesets found differentially expressed between pluripotent and differentiated samples (FDR threshold  = 0.1; log_2_ fold change threshold  = 1). Resultant probesets mapped to gene identifiers using the annotation packages in BioConductor.

### Chemotaxis assay

Chemotaxis was assessed using transwell chambers as previously described for cellular motility assays [16]. Costar Transwell 24-well plates exhibiting 5-µm pore size were used for all motility assays (Cambridge, MA). Briefly, for ES cells migration assays the transwells were immersed in gelatine solution overnight (0.1% in PBS) and rinsed in PBS. Transwells were blocked in fetal calf serum (FCS)-containing ES cell medium or for MEF migration assays FCS containing DMEM for 30 min at 37°C/5% CO_2_ and washed in PBS. ES cells and MEF were cultured as described above harvested and resuspended in culture medium (ES cells  = 1×10^5^ cells/ml, MEF  = 1×10^4^ cells/ml), and 100 µl of this suspension was added to the transwell plates onto a preformed chemotactic gradient (CXCL12, ES cells  = 10 ng, MEF  = 30 ng; CXCL10, ES cells  = 100 ng; CXCL16, ES cells  = 10 ng) and incubated overnight at 37°C/5% CO_2_. In all experiments there was no evidence of differential plating with varying conditions; chemotaxis was presented as a ratio with or without the chemokine. The transwells were then washed gently in PBS, and cells were removed from within the transwell using a dry cotton bud followed by two washes in PBS. This washing procedure was repeated twice. The transwells were stained with crystal violet for 10 min, washed in water, and allowed to air dry. Cells present on the underside of the transwell (i.e., migrated cells) were counted by microscopy. The number of cells on the bottom of the plate (i.e., cells that had migrated through the pores and become detached from the transwell) was also counted. P values were calculated using unpaired Student's *t* test. All chemotactic experiments were performed at least three times with triplicates for each condition.

Inhibition studies were performed in the presence of 10 µM Diprotin A, (Sigma) for CD26, 10 µM AMD3100 (Sigma) for CXCR4, 10 µg/well mouse antibody to CXCL12, (R&D systems), or 0.1–10 µg/ml of the monoclonal antibodies specific for m5T4. Specificity of mAb m5T4 mediated inhibition of chemotaxis was validated using m5T4-IgFc fusion protein [58].

### ELISA for CXCL12

The concentration of CXCL12 in 3 day conditioned medium from undifferentiated (+LIF) or following 3 days differentiation (-LIF) of WT- and 5T4KO-ES cells was determined by murine CXCL12 specific ELISA (R&D systems).

### Flow cytometry

Cell surface detection of human and murine 5T4 was performed as previously described [15], [16]. Briefly, cells were trypsinized, washed twice in PBS and resuspended at 2×10^6^ cells/ml in FACS buffer, (0.1% sodium azide, Sigma; 0.2% bovine serum albumin, Sigma; in PBS). Cells were labeled with antibodies at 4°C for 1 hour using monoclonal antibody (mAb) anti-SSEA-1-PE (phycoerithrin,) or mouse IgM isotype-PE control at 2 µg/ml (Santa Cruz)); rat anti-mDPIV(CD26)-PE, 5 µg/ml (R&D systems) or rat IgG_2A_ isotype control-PE 5 µg/ml (R&D systems); mAbs recognizing m5T4: 9A7 (rat IgG_2a_ [58] 20 µg/ml) and/or B3F1 (mouse IgG_2a_, 1 µg/ml), B5C9 (mouse IgG_1_ 10 µg/ml); B1C3 (mouse IgG_2a_ 10 µg/ml); P1C9 (mouse IgG_2b_ 1 µg/ml) and P1H10 (mouse IgG_2b_ 10 µg/ml); and their respective isotype controls rat IgG_2a_, mouse IgG_1_, mouse IgG_2a_ and mouse IgG_2b_ (eBioscience). Secondary antibodies were PE-donkey anti rat, 1 µg/ml (eBioscience); PE-goat anti-mouse, 1 µg/ml (DAKO). After washing twice in FACS buffer cells were fixed in 300 µl 1% *p*-formaldehyde in PBS. In situ immunoflurescence was usually performed for CXCR4 detection as the molecules can be sensitive to trypsin treatment.

### Modulationof 5T4 expression by mAbs

5×10^5^ B16m5T4 cells were incubated with either 1 µg/ml P1C9, 10 µg/ml B1C3, 10 µg/ml mouse IgG in DMEM supplemented with 10% FCS or media only at 37°C overnight. Cells were washed twice with FACS buffer and all of the following steps were performed in 0.1% sodium azide on ice. Cells were washed twice with FACS buffer and incubated with either biotinylated B3F1 (2 µg/ml), biotinylated B5C9 (25 µg/ml) or FACS buffer at 4°C for 45 mins. Cells were then washed twice and labeled with streptavidin PE (BD, 1∶100) at 4°C for 45 mins. Following two washes, cells were fixed in 1% *p*-formaldehyde in PBS and analyzed using a Becton Dickinson FACScan. 10,000 events were acquired and the data obtained was analyzed using WinMIDI (version 2.8) software.

### Quantitative PCR, (qPCR)

For each RNA sample, 2 µg of total RNA was reverse transcribed according to manufacturer's instructions, (Promega). Murine primers were designed using Primer Express 2.0 (read 5' to 3'; forward-F, reverse-R):

m5T4-F: agctcttcggtaccctcgtc,

m5T4-R: gttgcggttcacgcactta,

mCD26-F: ggcaatttgtaaaaatgggatt,

mCD26-R: aggttacataccctccatatgacc,

mCXCL12-F: tccaaattccccagcaga,

mCXCL12-R: ctgaacccatcgctgcttagac,

mCXCR4-F: caggacctgtggccaagttctt,

mCXCR4-R: agctgaggatcacggctagctt.

SybrGreen qPCR reactions were performed in MicroAmp optical 384-well reaction plates, (Applied Biosystems). Amplifications were carried out using a 7900 ABI Prism thermocycler, (Applied Biosystems) and amplification analyzed with SDS 2.1 software, (Applied Biosystems). Melt curves (derivative of fluorescence intensity versus temperature) were made and inspected to ensure that only one peak, indicating one amplicon product, was produced. Amplification efficiencies for each primer pair were determined by constructing standard dilution curves (mean Ct of triplicate reactions plotted against cDNA mass), with cDNA inputs of 1 ng, 5 ng, 10 ng, 50 ng and 100 ng per reaction and calculating *r* ^2^ values and gradients of linear regression lines. For testing of relative gene expression, 10 ng cDNA was used per reaction

### SDS-Polyacrylamide Gel Electrophoresis (PAGE) and Western Blotting

Samples were prepared in non-reducing PAGE loading buffer (Thermo Fisher), heated to 100°C for 3 minutes and loaded on to a preformed 10% or 4-15% gradient gel (BioRad) and run in Lamelli buffer (25 mM Tris-base, 192 mM glycine, 0.1% SDS). Western transfer to PVDF membranes used a BioRad Mini-PROTEAN Tetra cell system. For western probing, primary antibody concentrations were: Polyclonal rabbit anti-CXCR4, (1 µg/ml) (Abcam) and appropriate concentrations of the different mouse 5T4 specific monoclonal antibodies. For the detection of phosphorylated and/or intracellular proteins cells were lysed in M-PER supplemented with protease and phosphatase inhibitor cocktails, (Thermo Fisher). The compounds PD98059 (50 µM), LY294002 (50 µM) (both Cell Signaling Technology) or AMD3100 (10 µM) were applied to cells for 1 hour prior to CXCL12 (12.5 nM) or Phorbol 12-Myristate 13-Acetate, (PMA) (50 nM) (Sigma) stimulation in order to inhibit MEK1, PI3 kinase or CXCR4 respectively. Primary antibodies used as per suppliers instructions were ERK1/2, phospho-ERK1/2 (Thr202/Tyr204), (Cell Signalling Technology). Following secondary antibody labeling using appropriate HRP conjugates, (AbSerotec) hybridizing bands were detected using SuperSignal West Dura, (Thermo Fischer).

### Immunofluorescence in situ

These studies were performed using methods described previously [63], with cells grown on 24 well glass bottomed plates, (Iwaki, supplied through Jencons) coated with 0.1% gelatine. Primary antibodies used were rabbit anti-CXCR4, (5 µg/ml) (Abcam), rat-anti-DPIV (5 µg/ml), (R&D), mAb anti-m5T4, 9A7 (20 µg/ml), B5C9 or B3F1 (5 µg/ml) or appropriate isotype controls. Secondary detection was performed by incubation for 1 hour at 4°C with species or IgG sub-class specific Alexa Fluor conjugated secondary antibodies (Invitrogen) (6 µg/ml) as appropriate for multiple antigen detection. Labeling of the endoplasmic reticulum and Golgi apparatus was performed using C_6_-Ceramide or Endotracker, (Invitrogen, Molecular Probes) as per manufacturer's instructions. Labeling of lipid rafts was performed using cholera toxin subunit B conjugated to FITC, 1 µg/ml (Sigma) for 30 minutes at room temperature after secondary antibody labeling. F-Actin was labeled using Phaloidin-633 Alexa Fluor (Invitrogen). Inhibition of the Golgi, actin cytoskeleton and microtubule network was performed by overnight incubation of MEF with optimized concentrations of 3.57 µM Brefeldin FA, 985 nM Cytochalasin D or 332 nM Nocodazole respectively. Cells were fixed following washout at 0, 30 minutes and 1 hour and processed as above for immunofluorescence. Cytoskeleton disruption following 24 hours treatment with cytochalasin D was achieved as no polymerized actin filaments were detectable (data not shown). Likewise, Golgi disruption following brefeldin A treatment was confirmed by immunofluorescence detection of sphingolipids using BODIPY labeled NBD C6 ceramide (data not shown). Microtubule disruption following nocodazole treatment was confirmed by immunofluorescence detection using an antibody against β-tubulin (data not shown). In some experiments, MEF were seeded and treated with 332 nM nocodazole for 18 hours and following washout, cells were incubated in growth medium or growth medium with mAb B1C3 and P1C9 (10 µg/ml) or mIgG (10 µg/ml) for 3 or 6 hours.

Cells were imaged on a Zeiss Axiovert 200 M with a plan-fluar ×100 1.45NA objective lens and a Roper Cascade EMCCD 512B camera. Illumination was achieved using a 300W Xenon system, (Sutter) which presented the system as an even field of illumination in addition to the appropriate neutral density and Schott filters to modulate the light source. Wavelength selection was achieved using external filter wheels, (Applied Scientific Instrumentation) and the ET-Sedat set, (Chroma). Data sets were captured with an axial resolution of 100 nm using the MS-2000 stage (Applied Scientific Instrumentation) and a lateral resolution of 0.1645 microns per pixel. The system was full controlled and automated via the FRAP-AI software, (MAG Biosystems/Metamorph). All of the data sets were deconvolved using Huygens (Scientific Volume Imaging) after which visualization and analysis was carried out using Imaris, (Bitplane). Deconvolved images were assessed utilizing the ImarisColoc software (Bitplane) in manual mode. A 2D scatter plot showing intensity pairs in the image was thresholded to include only co-localized points in the three dimensional volume. This data was then extracted to a separate channel containing three dimensional co-localized points only.

For each in situ immunofluorescence investigation, a minimum of 50 cells per experimental condition were examined in at least duplicate. In addition a more detailed quantitative analysis (as above) of the patterns of expression (e.g. intracellular versus membrane) was performed on between 10–25 cells and representative images are presented.

### 5T4 constructs

A series of 5T4 constructs were built for this study and cloned into pCMVα, and the retroviral vector SFβ91 [64] containing a cDNA cassette eGFP under the transcriptional control of an IRES, (Clonetech). Chimeric constructs of mouse 5T4/CD44 molecules with reciprocally exchanged TM and cytoplasmic domains were engineered. CD44 molecules are 80–95 kDa transmembrane glycoproteins expressed on a variety of normal cells as well as some tumors where particular spliced forms have been associated with increased metastasis [65]. E1/E3 replication deficient recombinant adenoviral vectors were constructed by cloning of the m5T4 or h5T4 cDNA into the adenoviral shuttle vector pΑdlox [66]. GFP control adenoviral vector was generated by the sub-cloning of the eGFP cDNA into the pAdlox vector. Recombinant adenoviral particles, (hereafter termed RAd-m5T4, RAd-h5T4 and RAd-GFP) were generated by co-transfection of CRE8 cells with the pAdlox vector and adenovirus C5 DNA as described [66]. High-titre stocks were prepared by double cesium chloride density gradient separation and titred as previously described [63]. Viral stocks were found to be free of replication-competent adenovirus using a supernatant rescue assay using HeLa cells able to detect 1 replication-competent virus within 10^9^ recombinant viruses [63]. A multiplicity of 30 infectious units per cell led to 100% of cells expressing m5T4 or h5T4 or GFP at 48 hours as assessed by FACS and when other biological assessments were made.

### Statistical analysis

Quoted errors refer to standard errors of the mean. Statistical significance was calculated by either two-tailed unpaired Student t-test or ANOVA test as appropriate.
